# Positive Effects of Organic Substitution in Reduced-Fertilizer Regimes on Bacterial Diversity and N-Cycling Functionality in Greenhouse Ecosystem

**DOI:** 10.3390/ijerph192416954

**Published:** 2022-12-16

**Authors:** Na Sun, Liying Wang, Yanxin Sun, Hong Li, Shangqiang Liao, Jianli Ding, Guoliang Wang, Linna Suo, Yanmei Li, Guoyuan Zou, Shaowen Huang

**Affiliations:** 1 Institute of Plant Nutrition, Resources and Environment, Beijing Academy of Agriculture and Forestry Sciences, Beijing 100097, China; 2 Institute of Agricultural Resources and Environment, Hebei Academy of Agriculture and Forestry Sciences, Shijiazhuang 050051, China; 3 Institute of Biotechnology, Beijing Key Laboratory of Agricultural Genetic Resources and Biotechnology, Beijing Academy of Agriculture and Forestry Sciences, Beijing 100097, China; 4 Institute of Agricultural Resources and Regional Planning, Chinese Academy of Agricultural Sciences, Beijing 100081, China

**Keywords:** soil aggregation, C/N ratio, environmental pollution, swine manure, maize straw, bacterial diversity

## Abstract

Conventional fertilization in the greenhouses of North China used excessive amounts of chemical and organic fertilizer, resulting in soil degradation and severe agricultural non-point source pollution. A nine-year study was conducted on a loamy clay soil in Shijiazhuang, Hebei province, to investigate the effects of reduced-fertilizer input regimes on soil property, bacterial diversity, nitrogen (N) cycling and their interactions. There were four treatments, including high organic + chemical fertilizer application rate and three reduced-fertilizer treatments with swine manure, maize straw or no substitution of 50% chemical N. Treatments with reduced-fertilizer input prevented soil salinization and acidification as in local conventional fertilization after being treated for nine years. In comparison to chemical fertilizer only, swine manure or maize straw substitution maintained higher nutrient availability and soil organic C contents. Fertilizer input reduction significantly increased bacterial richness and shifted bacterial community after nine years, with decisive factors of EC, Olsen P and C/N ratio of applied fertilizer. Soil chemical characteristics (EC, pH and nutrients), aggregation and C/N ratio of applied fertilizer selected certain bacterial groups, as well as N-cycling functions. Reduced-fertilizer input decreased the potential nitrification and denitrification functioning of bacterial community, but only in organic substitution treatments. The results of this study suggested that fertilizer input reduction combined with organic C input has potential in reducing non-point source pollution and increasing N-use efficiency in greenhouse vegetable production in North China.

## 1. Introduction

Excessive chemical fertilizer (N in particular) application has been the main reason for severe agricultural non-point source pollution in China since the 1990s, due to the reasons of yield as a priority and lack of agricultural extension services in the small-scale farming system [1,2,3]. As the second-highest contributor, vegetable production discharge was the main reason for slow reductions in agricultural non-point source pollution, resulting in large environmental problems (such as greenhouse gas emission and nitrate pollution) via N loss to atmosphere or entry to water bodies in China [4]. Meanwhile, continuous over-application of chemical fertilizer triggered soil degradations, including soil acidification, soil salinization, declined fertility microbial diversity and an unstable soil micro-ecosystem. Plant productivity, in view of yield and quality, was consequently reduced [1]. In addition, organic wastes (mainly animal waste and crop straw) that have not been recycled to agricultural land as before were improperly handled via burning and direct discharge, inducing severe air and water pollution [5]. Over-fertilization has limited the sustainable and environmental-friendly development of greenhouse vegetable production in China.

In the last decade, an agreement has been reached on reducing total fertilizer input and increasing organic fertilizer usage on boosting soil health and plant productivity [6,7]. Compared to chemical fertilizer, organic manure application and crop residue return could maintain soil fertility and crop yield with enhanced soil organic matter and mineral nutrients [5]. Huang et al. [7] investigated fertilizer application in 578 greenhouse vegetable production plots all over China and recommended a chemical fertilizer reduction potential of 34.8–67.1% from 1355 kg ha^−1^ (N + P_2_O_5_ + K_2_O) based on soil nutrients, target yield and fertilizer application rate. Zhang et al. [8] reported that a 40% reduction in chemical fertilizer input (from N-P_2_O_5_-K_2_O of 858-594-1284 kg ha^−1^) can increase N uptake and tomato yield. In the meantime, a combination of chemical and organic fertilizer was recommended for soil ecosystem restoration [9]. A 40–50% substitution of chemical fertilizer with organic fertilizer was suggested appropriate in maintaining high yield, while improving soil quality, soil carbon (C) content and soil aggregate stability [7,10]. Current advice on proper fertilizer reduction is based on soil property, which was insufficiently accurate and often has a delay of several years in response to agricultural practices [11].

In agricultural ecosystems, anthropogenic interference, for example, fertilization and nutrient source selection, has profound effects on soil property, soil microbial diversity and ecosystem service [12]. Soil microbes are the most active and sensitive components in a soil ecosystem and are highly involved in organic matter decomposition, nutrient cycling and soil aggregation [13,14]. Soil microbes have rapid response to agricultural management and soil property alteration, while exerting an influence on soil property. In the meantime, microorganisms and soils could affect aboveground plants via material cycling and energy flow in the terrestrial ecosystem [15,16]. Soil microbial community was, thereby, viewed as an important indicator of soil environment, with growing research has focused on the interaction between soil bacterial functional traits and soil properties in distinct ecosystems, from field to global scale [17,18,19]. However, previous studies characterized soil bacterial community as affected by various fertilization regimes on mostly cereal crops, such as rice and rice–wheat rotation on paddy soils [9,18,20], maize–wheat rotation on Calcaric Fluvisol soil [19], maize, wheat and soybean on black soil [21,22,23]. Up to date, the knowledge is still limited on bacterial responses to distinct fertilizer input reduction practices in greenhouse vegetable production [24], especially on the mechanisms of bacterial community in the regulation of soil ecosystem service.

Due to the slow change in soil property, we adopted a long-term in situ study with distinct fertilizer input reduction regimes in a greenhouse to (1) identify the bacterial taxonomy and N-cycling functional traits under distinct fertilizer reduction regimes; (2) address the mechanism in soil bacterial responses and adaptations to distinct fertilizer reduction regimes.

## 2. Materials and Methods

The experiment was initiated in a plastic solar greenhouse (384.0 m^2^, 48.0 m × 8.0 m) from August 2009 on a loamy clay soil in Shijiazhuang, Hebei Province, China. The experimental location had low-fertility soil with properties (0–20 cm) as follows: electrical conductivity (EC) of 185.4 μS cm^−1^, pH of 8.0, soil organic C (SOC) content of 5.3 g kg^−1^, soil nitrate nitrogen (N) content of 18.3 mg kg^−1^, available phosphorus (P) content of 6.2 mg kg^−1^ and available potassium (K) content of 98.2 mg kg^−1^ before planting [25].

A randomized complete block design with four fertilization treatments and three replications was adopted. Each block (2.4 × 6 m) consisted of four rows with the middle two as sampling rows. Each row contained 20 plants with a spacing of 0.3 m between plants. Additional border rows along the eastern and western sides of the greenhouse were planted. A crop rotation of cucumber (*Cucumis Sativus* L., from February to July) and tomato (*Lycopersicon esculentum* Mill, from August to February in the following year) was applied in the greenhouse. Irrigation, pest control and regular management of the crops were the same for all treatments.

### 2.1. Fertilizer Treatments

Annual NPK inputs from different fertilizer sources for each treatment are listed in Table 1. Local conventional fertilization (LCF) is considered as control, with a total annual NPK input of 2100, 790 and 1520 kg ha^−1^, while the other three treatments had annual NPK input of 1050, 229 and 934 kg ha^−1^ calculated according to soil nutrient status and target yields of cucumber and tomato (Table 1). Equal amounts of total NPK were set up for these three treatments as follows: (1) 50% of chemical N and 50% of swine manure N (2/4 CN + 2/4 MN); (2) 50% of chemical N and 50% of maize straw N (2/4 CN + 2/4 SN); (3) 100% chemical N (4/4 CN). Total P and K input in these three treatments were brought to equal amount with chemical fertilizer if in shortage. The annual total C input was 4491, 22,690, 0 and 7699 kg ha^−1^ in 2/4 CN + 2/4 MN, 2/4 CN + 2/4 SN, 4/4 CN and LCF, respectively (Rong, 2018). Chemical fertilizers applied were urea (46% N), calcium superphosphate (16% P_2_O_5_) and potassium sulphate (51% K_2_O) for all treatments. The NPK contents were 16.7, 2.8 and 4.3 g kg^−1^ FW in commercial swine manure (water content of 33.2%), and 7.5, 0.4 and 5.0 g kg^−1^ FW in maize straw (water content of 13.7%), respectively. Maize straw was chopped and applied 20–25 cm beneath ground level before planting. All swine manure, 20% of the chemical N, 100% chemical P and 40% of chemical K were broadcast to soil before planting, followed by a rotary plough. The rest of the chemical N and K was applied four times during the growing season.

### 2.2. Soil Property Analysis

Soil samples (0–20 cm) were taken on 25 December 2018 and then air-dried for the determination of soil properties: EC, pH, total N content, alkali hydrolysable N content, Olsen P content, available K content, organic C content and aggregate size distribution. Soil pH was determined at soil:water ratio (*w*/*w*) of 1:2.5 using a pH meter (model LAQUAtwin pH-11, Horiba, Kyoto, Japan). Soil EC was determined at soil:water ratio (*w*/*w*) of 1:5 using conductivity meter (model MP515 Shanghai Sanxin, Shanghai, China). Soil total N content was examined with a continuous flow analyzer (Auto Analyzer 3 System, SEAL Analytical GmbH, Norderstedt, Germany), while available K content was analyzed with an atomic absorption spectrophotometer (ZEEnit700P, Analytic Jena, Jena, Germany). Detailed information can be found in Sun et al. [26]. Alkali hydrolysable N content was determined according to Lu [27] and Olsen P content was determined according to Olsen et al. [28]. Soil organic C content was determined following Nelson and Sommers [29]. Soil aggregate size distribution was determined with wet-sieving method [30].

### 2.3. DNA Extraction, PCR Amplification and Sequencing Data Processing

Bacterial genomic DNA was extracted from 0.5 g fresh soil (0–20 cm, taken on December 25, 2018). Then, 16S rDNA gene was amplified in the hypervariable V3-V4 region with a pair of primers, 338F (5′-ACTCCTACGGGAGGCAGCAG-3′) and 806R (5′-GGACTACHVGGGTWTCTAAT-3′). The polymerase chain reaction (PCR) was first denatured at 95 °C (3 min), then went through 27 cycles of denaturing (95 °C for 30 s), annealing (55 °C for 30 s) and extension (72 °C for 45 s), with a final extension at 72 °C (10 min). A 20 μL mixed solution of buffer (TransStart FastPfu, 4 μL), dNTPs (2.5 mM, 2 μL), primers (5 μM, 0.8 μL), DNA Polymerase (0.4 μL), BSA (0.2 μL), template DNA (10 ng) and ddH_2_O was used for PCR reaction [31]. Purified amplicons were processed on an Illumina MiSeq PE300 platform (Illumina, San Diego, CA, USA) by Majorbio Bio-Pharm Technology Co. Ltd. (Shanghai, China). We deposited the gene sequences of all samples in the National Center for Biotechnology Information (NCBI) Sequence Read Archive (PRJNA818078). Fastp (https://github.com/OpenGene/fastp, version 0.20.0, accessed on 13 December 2022, HaploX Biotechnology, Shenzhen, China) and FLASH (http://www.cbcb.umd.edu/software/flash, version 1.2.7, accessed on 13 December 2022, University of Maryland, College Park, MD, USA) were used processing sequencing data for barcodes and primer trimming and low-quality read removal (<Q20) [32,33]. In total, 529,347 effective sequences were obtained for the tested soil samples (12 samples, 4 treatments with 3 replications). High-quality sequences (26,367 for each sample) were clustered into operational taxonomic units (OTUs) using UPARSE (http://drive5.com/uparse/, version 7.1, accessed on 13 December 2022, Robert Edgar, Tiburon, CA, USA) with a 97% similarity and then compared to database Silva (Release115, http://www.arb-silva.de, accessed on 13 December 2022, Marine Microbilogy and Jacorbs University, Bremen, Germany) with a confidence threshold of 0.7 using RDP classifier (http://rdp.cme.msu.edu/, version 2.2, accessed on 13 December 2022, Michigan State University, East Lancing, MI, USA) [34].

### 2.4. Data Analysis

Analysis of variance (ANOVA) was conducted for soil properties (EC, pH, soil nutrients, organic C and the proportion of aggregates), observed OTU and alpha diversity indices (Chao1, ACE, Shannon and Simpson) as a function of fertilizer treatments using the PROC ANOVA procedure in SAS (SAS Institute Inc., Cary, NC, USA). Means were compared using the Least Significant Difference (LSD) test (5% probability). Alpha-diversity of bacterial community was assessed using Mothur (http://www.mothur.org/, version 1.31.2, accessed on 1 November 2022), while beta-diversity was calculated using the Bray–Curtis dissimilarity metric [35]. Bacterial taxonomy of all four treatments was visualized in Venn diagram analysis in R and stacked-column diagram analysis in Origin 2021 (OriginLab Corporation, Northampton, MA, USA). Bacterial N-cycling functions were predicted using FAPROTAX and demonstrated in a stacked-column diagram via Origin 2021. Correlation between bacterial diversity, composition, soil property and N-cycling functions was analyzed using Spearman correlation analysis and then plotted with heatmaps in Origin 2021. A redundancy analysis (RDA) was performed for the correlation of multiple variations between soil characteristics and bacterial community composition (genus level) in CANOCO 5.0.

## 3. Results

### 3.1. Soil Property as Affected by Fertilizer Reduction Regimes

Fertilization regimes significantly affected soil properties after the nine-year treatment in 2018 (Table 2). Compared to the soil properties in 2009, all treatments had increased soil EC and SOC, decreased soil pH and accumulated soil nutrients (P and K). In 2018, excessive fertilizer application (LCF) accumulated high soil EC (1261 μs cm^−1^) and soil nutrient contents (total N, alkali-hydrolysable N, Olsen P and available K contents), induced soil acidification (pH of 5.4) and promoted shifting from fine particle (<0.25 mm) to coarse particles (0.25–2 mm), in comparison to treatments with reduced fertilizer application rates. With fertilizer input reduction, treatments (4/4 CN, 2/4 CN + 2/4 MN and 2/4 CN + 2/4 SN) had significantly lower soil EC and nutrient contents while maintaining a stable soil pH and aggregate distribution compared to LCF. With equal amounts of NPK input, fertilizer reduction with swine manure or maize straw substitution had higher soil nutrients and organic C levels and lower soil EC than chemical-fertilizer-only treatment after nine years, indicating a pronounced effect in improving soil quality.

### 3.2. Bacterial Community as Affected by Fertilizer Reduction Regimes

Bacterial richness (Chao1 and ACE) was more responsive to fertilizer treatments than diversity indexes (Shannon and Simpson). Fertilizer input reduction significantly increased OTU observed, Chao1 and ACE estimates (Table 3), indicating a restraining effect of excessive fertilizer application on bacterial richness. Equal NPK inputs with different N sources had no significant impacts on bacterial community richness after the nine-year treatment. The results suggested a significant positive effect of fertilizer reduction on bacterial richness.

**Figure 1:**
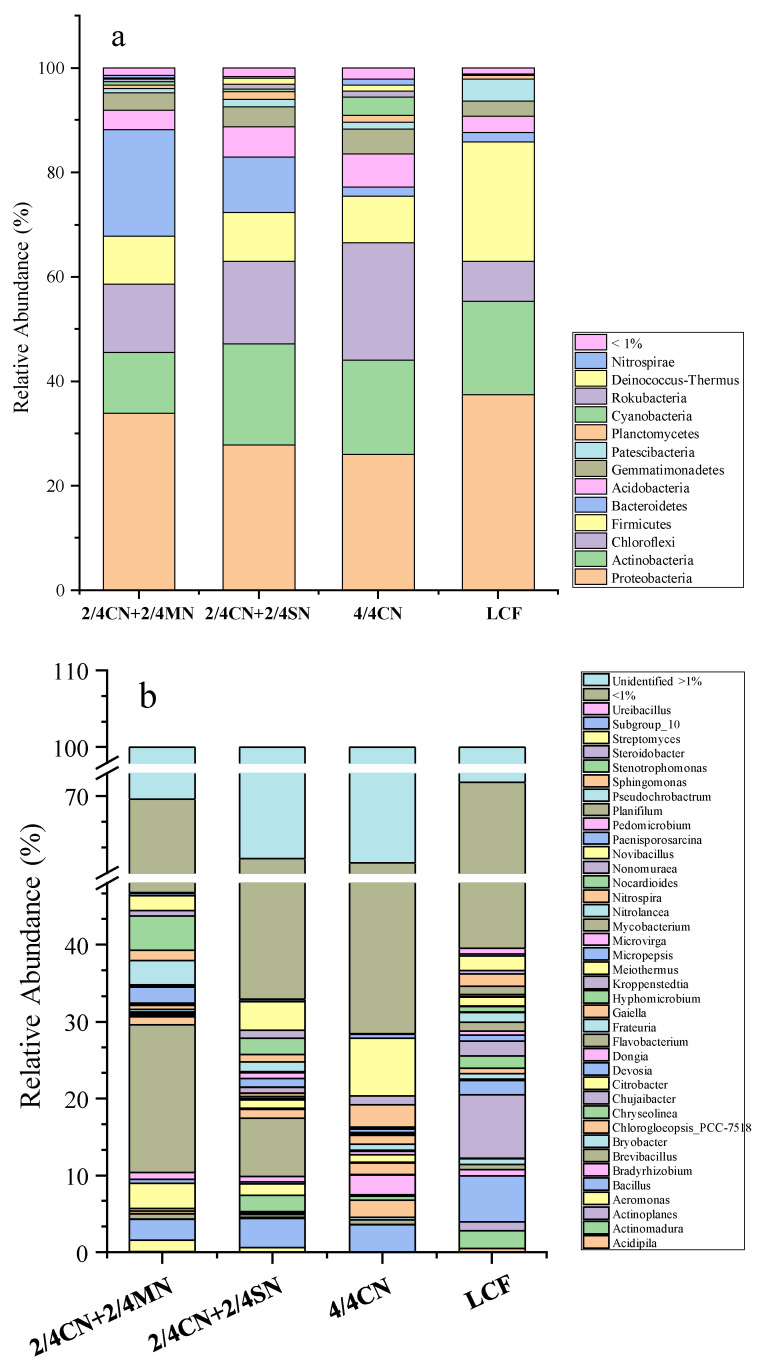
Community composition and clustering analysis of all treatments at phylum (**a**) and genus (**b**) levels. CN: chemical nitrogen; MN: swine manure nitrogen; SN: maize straw nitrogen; LCF: local conventional fertilization.

The taxonomic composition of the four treatments was analyzed on phylum and genus levels (Figure 1). There were 13 phyla with relative abundance > 1% in at least one sample (Figure 1a). The dominant phyla across all four treatments were Proteobacteria (25.02–34.56%), Actinobacteria (11.59–21.05%), Chloroflexi (7.82–21.68%), Firmicutes (8.54–23.00%) and Bacteroidetes (1.69–20.50%), accounting for 77.99–88.22% of the bacterial sequences. Fertilization regimes significantly affected the relative abundances of the five dominant phyla, as well as Patescibacteria, Cyanobacteria, Rokubacteria and Nitrospirae. Fertilizer input reduction from LCF significantly reduced relative abundances of three phyla (Proteobacteria, Firmicutes and Patescibacteria) and increased relative abundances of five phyla (Chloroflexi, Bacteroidetes, Nitrospirae, Rokubacteria and Cyanobacteria). Among these five phyla, Bacteroidetes were enriched only with organic N substitution in 2/4 CN + 2/4 MN and 2/4 CN + 2/4 SN while Cyanobacteria were enriched only in 4/4 CN.

Bacterial communities of the four treatments comprised a total of 77 genera (relative abundance > 1.0% in least one sample), accounting for 67.82–77.11% of the total sequences (Figure 1b). Further, 21 identified genera were significantly affected by fertilizer input reduction, accounting for 10.12–38.12% of the total sequences. Fertilizer input reduction from LCF significantly decreased relative abundances of 11 genera, including *Actinomadura*, *Actinoplanes*, *Bacillus*, *Bradyrhizobium*, *Bryobacter*, *Chujaibacter*, *Devosia*, *Hyphomicrobium*, *Micropepsis*, *Nocardioides* and *Planifilum*. Certain genera were enriched in distinct nutrient sources, such as *Aeromonas*, *Citrobacter*, *Flavobacterium*, *Pseudochrobactrum* and *Stenotrophomonas,* in 2/4 CN + 2/4 MN and 2/4 CN + 2/4 SN, *Chryseolinea* and *Nonomuraea* in 2/4 CN + 2/4 SN and *Nitrospira* and *Sphingomonas* in 4/4 CN.

**Figure 2:**
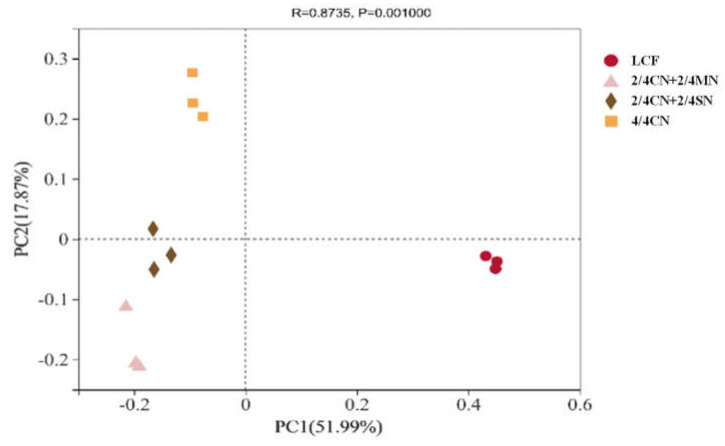
Principal coordinate analysis of bacterial community (genus) of all treatments. CN: chemical nitrogen; MN: swine manure nitrogen; SN: maize straw nitrogen; LCF: local conventional fertilization.

PCoA revealed the impacts of long-term fertilization regimes on bacterial community (genus level, Figure 2). The first two principal coordinates represented 69.86% of the total variation in bacterial community. LCF was distinct with other treatments on PC1, suggesting a profound impact of fertilizer input reduction on bacterial community. Organic N substitution in 2/4 CN + 2/4 MN and 2/4 CN + 2/4 SN resulted in their separation from 4/4 CN on PC2.

### 3.3. Potential Bacterial N-Cycling Functioning

**Figure 3:**
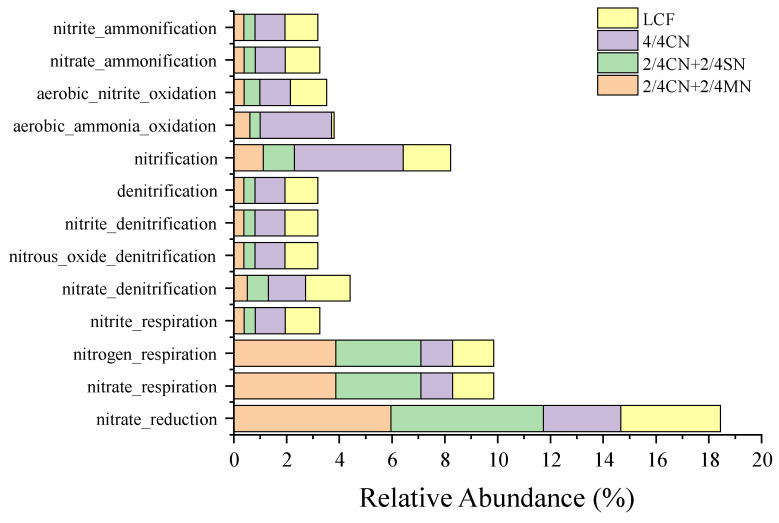
Potential N-cycling functioning of all treatments predicted by FARPOTAX. CN: chemical nitrogen; MN: swine manure nitrogen; SN: maize straw nitrogen; LCF: local conventional fertilization.

A succession of 13 N-cycling functions of bacterial community was predicted by FAPROTAX (Figure 3). These N-cycling functions were mainly related to nitrification, denitrification and inorganic N ammonification processes, with ‘nitrification’ (1.21–3.86%), ‘nitrogen respiration’ (1.21–3.86%), ‘nitrate respiration’ (1.21–3.86%) and ‘nitrate reduction’ (2.94–5.96%) as the main functions. Relative abundances of ‘nitrogen respiration’, ‘nitrate respiration’ and ‘nitrate reduction’ were significantly increased after fertilizer input reduction, but only when substituted for 1/2 organic N. Thus, 4/4 CN had significantly higher abundances of ‘aerobic nitrite oxidation’ and ‘nitrification’ than all other treatments. All other functions (mainly related to denitrification and inorganic N ammonification) were significantly higher in LCF and 4/4 CN than in 2/4 CN + 2/4 MN and 2/4 CN + 2/4 SN.

## 4. Discussion

### 4.1. Fertilizer Reduction Improved Soil Quality after the Nine-Year Application

Continuous over-fertilization can cause soil acidification and salinization [36,37]. A coexistence of soil acidity (decreased by 0.24–1.06 units) and salinity (increased by 0.24–0.68 ms cm^−1^) from excessive urea application (up to 1200 kg ha^−1^) was reported in a greenhouse-grown lettuce [38]. According to Kingery et al. [39], soil EC of 4/4 CN and LCF in this study exceeded the tolerance limit of 0.4 mS cm^−1^ for most vegetable crops. Even with a high proportion of swine manure input, LCF induced soil salinization and acidification after the nine-year application. The given environmental conditions of high temperature, high humidity and frequent irrigation, while lacking leaching from natural precipitation in the greenhouse, may have accelerated this process in the top layer of soils [40,41]. The three treatments with reduced fertilizer application rates avoided soil acidification and salinization, among which swine manure and maize straw substitution had higher soil nutrient availability (N and P), higher organic C contents and lower soluble salt contents than pure chemical fertilizer treatment after nine years, consistent with conclusions from previous studies [42,43,44]. Soils that received only chemical fertilizer in the long term could not maintain soil nutrient and organic C levels [45,46], as N fertilizer enhanced microbial activity and stimulated a fast decomposition of soil organic C with no exogenous C addition [1,47]. On the other hand, compared to pure chemical fertilizer treatment 4/4 CN, 2/4 CN + 2/4 MN and 2/4 CN + 2/4 SN had partial organic N and P, which had a longer nutrient releasing time due to decomposition [48,49].

A high application rate of swine manure in LCF increased macroaggregate proportion and, consequently, soil permeability after the nine-year application in this study, which was consistent with the conclusion of organic fertilizer application promoting the shift from microaggregate to macroaggregate particles in previous studies [50,51]. Soil aggregate distribution was not significantly different in the three treatments with equal NPK input after nine years in this study. Previous studies reported different results, that organic fertilizer had a promoting effect on macroaggregates in a long-term maize cropping field and in a rice–rape cropping system [52,53]. However, this promoting effect varied by crop and soil type, which possibly resulted in the differences between this and previous studies [11]. Furthermore, changes in soil aggregation from fertilization regimes occur slowly and the effects of organic N substitution may become significant in the following years.

### 4.2. Bacterial Diversity and Composition to Altered Soil Properties from Fertilization

#### 4.2.1. Increased Bacterial Richness

**Figure 4:**
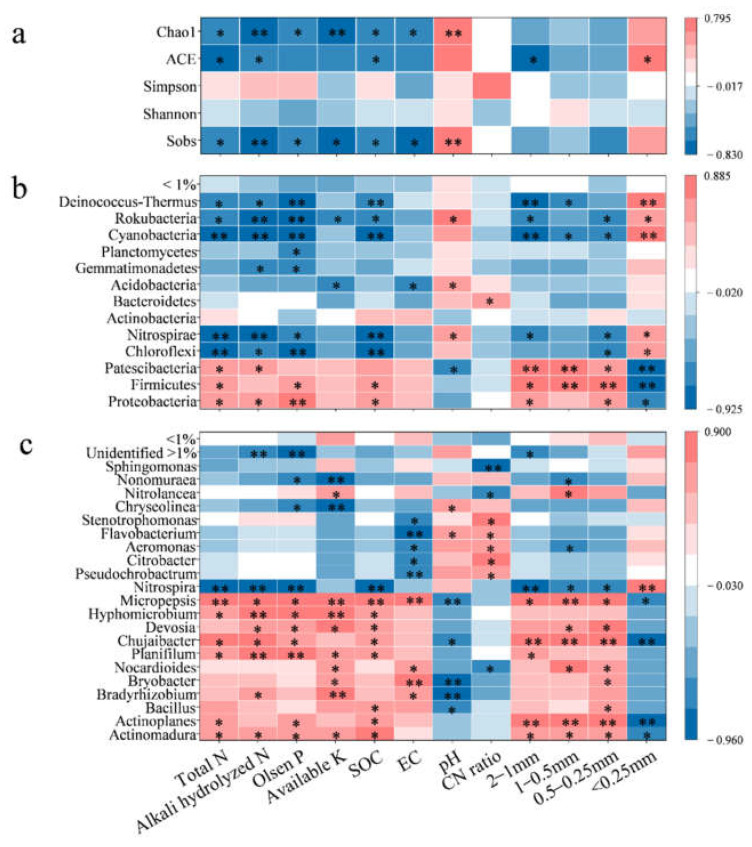
Spearman correlation analysis between soil properties and bacterial diversity (**a**) and composition (on phylum (**b**) and genus (**c**) levels). * indicates significant correlation at *p* < 0.05, while ** indicates extremely significant correlation at *p* < 0.01.

Bacterial richness generally has a positive response to C and N input in organic fertilizer [54]. In this study, bacterial richness was significantly correlated with soil chemical properties, such as EC, pH, nutrients (NPK) and organic C (Figure 4a). A previous study reported similar results of restrained bacterial reproduction and decreased bacterial richness due to a substantial accumulation of soil nutrients, soil salinization and acidification [55]. Soil aggregation (<0.25 mm and 1–2 mm) also played an important role in bacterial richness, demonstrating significant correlations with ACE. Previous studies reported that soil aggregation affects bacterial diversity via regulating soil habitat architecture and nutrient resources [56], with a diverse bacterial community in microaggregates [57] and a negative correlation between macroaggregates (0.25–2 mm) and bacterial alpha diversity [58]. This is consistent with the result in this study. Bacterial diversity did not respond to organic material substitution and might be related to the similar soil aggregate distribution between 4/4 CN, 2/4 CN + 2/4 MN and 2/4 CN + 2/4 CN. As soil biota can reproduce in diverse soil conditions that are seldomly optimal, organic material substitution may have a lagged effect on bacterial diversity, especially in soils with a large proportion of microaggregates [59]. Differences in bacterial diversity between 4/4 CN, 2/4 CN + 2/4 MN and 2/4 CN + 2/4 CN might be significant in the following years.

#### 4.2.2. Potential Keystones of Bacterial Community Selected by Altered Soil Properties

Core bacteria can enhance protection from abiotic factors and maintain ecosystem multifunction; thereby, they were often considered key to improving agricultural sustainability and productivity [12]. The relationships between bacterial abundance and soil properties are observed with Spearman correlation analysis (Figure 4b,c). Chloroflexi, Nitrospirae, Cyanobacteria, Rokubacteria and Deinococcus-Thermus were in negative correlations with soil nutrients (N and P) and organic C (*p* < 0.05) and positive correlations with soil pH (Figure 4b). These phyla were enriched after fertilizer input reduction, indicating that they were prone to less-fertile soil. For instance, previous studies reported Chloroflexi and Nitrospirae in less-fertile soil [60,61], with abundances decreased with increasing organic fertilizer application [62,63]. In addition, reduced soil nutrients may have stimulated the enrichment of certain bacteria and their given functions in biogeochemical processes. Cyanobacteria and Nitrospirae abundances were the highest in 4/4 CN with the lowest soil N content, possibly for their potential in N fixation and nitrification, respectively [64,65]. On the contrary, Proteobacteria, Firmicutes and Patescibacteria were positively correlated with soil nutrients and organic C in this study. Proteobacteria and Firmicutes are copiotrophic bacteria that are involved in organic substrate degradation and C/N cycling [23,63] and were enriched in a high soil nutrient environment [63,66]. Patescibacteria abundance was reported in positive correlation with soil nitrate content and N-fertilizer input in a previous study [67] and this study. Soil pH also contributed to the altered bacteria abundances. Nitrospirae and Patescibacteria also responded to soil pH in positive and negative correlations, respectively, which was consistent with previous studies [68,69].

A similar pattern was observed at the genus level as well. Bacteria abundances that decreased after fertilizer input reduction were positively correlated with soil nutrients, organic C and EC, and negatively correlated with soil pH (Figure 4c), such as *Actinomadura*, *Actinoplanes*, *Bacillus*, *Bradyrhizobium*, *Bryobacter*, *Chujaibacter*, *Devosia*, *Hyphomicrobium*, *Micropepsis*, *Nocardioides* and *Planifilum* (Figure 4c). Their genera were generally tolerant to acidity and salinity and more adapted to fertile soil with high organic C content. Similar results were reported in previous studies. The relative abundances of bacteria *Actinomadura*, *Actinoplanes*, *Bacillus*, *Devosia* and *Planifilum* that decreased with reduced fertilizer application rate were reported in previous studies [63,70]. *Bradyrhizobium* was enriched under salinity stress conditions [71]. Cowpea plants inoculated with *Bradyrhizobium* in combination with *Actinomadura* and *Bacillus* (plant-growth-promoting bacteria) under salt stress showed greater oxidative protection with increased antioxidase activity and lower H_2_O_2_ contents in plant tissue [72].

Soil aggregates were essential to biogeochemical cycling for they were the key functional units in the soil ecosystem [53]. Long-term organic fertilizer application was reported to promote soil macro-aggregation via increasing organic matter as the binding agent for soil particles [53,73] and, consequently, affected bacterial community [74]. Bacterial phyla and genera that were responsive to chemical properties (nutrients, EC, pH and SOC) also responded to soil aggregation (Figure 4). For those adapted to fertile soil with low pH, their abundances were increased with a high proportion of macroaggregates (0.25–2 mm). On the contrary, bacterial abundances increased after fertilizer input reduction and were favored for microaggregate < 0.25 mm. Feng et al. [75] and Ye et al. [74] reported copiotrophic bacteria Proteobacteria in macroaggregates (0.25–2 mm) under long-term fertilization or organic fertilizer, which was consistent with the results in this study. Soil aggregates varied in organic matter, nutrient availability and oxygen diffusion capacity [75]. Macroaggregates had high nutrient, labile C and oxygen contents that supported copiotrophic bacteria reproduction, while oligotrophic bacteria were gathered in microaggregates with fewer resources [25,76]. However, this is not always the case. Zheng [77] reported contradictory results of increased abundance of Chloroflexi, and decreased Proteobacteria abundance within macroaggregates were also reported in aged apple orchards. The different ecosystems, background soil conditions and fertilization durations might be the possible causes for the contradictory results.

### 4.3. Potential Keystones of Bacterial Community Selected by C/N Ratio of Input Fertilizer

Different organic materials had varied C/N ratios, with 4.3 for 2/4 CN + 2/4 MN, 21.6 for 2/4 CN + 2/4 SN and 3.7 for LCF in this study. Compared to decomposed organic fertilizer, maize straw contained a high content of recalcitrant C, such as cellulose, hemicellulose and lignin. Consequently, soil C/N ratio in 2018 was lower in 4/4 CN (6.7) than the other three treatments (8.7–9.8) in 2018. According to Spearman correlation analysis, there were bacteria that responded to C/N ratio of the input fertilizer, rather than soil physicochemical properties, including phyla of Bacteroidetes and genera of *Flavobacterium*, *Aeromonas*, *Citrobacter*, *Pseudochrobactrum* and *Stenotrophomonas* (Figure 4b,c). These bacteria were previously reported to be involved in organic matter degradation and nutrient cycling, due to the addition of organic materials [78]. Bacteroidetes are known for their ability to degrade complex organic matter, including starch, proteins, xylan, cellulose and chitin [9,79], which supported their enrichment in 2/4 CN + 2/4 MN and 2/4 CN + 2/4 SN. A similar situation of increased *Flavobacterium*, *Aeromonas*, *Citrobacter*, *Pseudochrobactrum* and *Stenotrophomonas* abundances after fertilizer input reduction with partial organic material substitution was also observed. Previous studies reported that *Flavobacterium*, *Citrobacter* and *Stenotrophomonas* degrade organic matter, are involved in N cycling (such as promoting nutrient cycling and enhancing soil N) and are enriched when organic materials were introduced into the soil [45,80,81,82], which explained their higher abundances in treatments with continuous C input (2/4 CN + 2/4 MN and 2/4 CN + 2/4 SN) in this study. A higher C/N ratio in the input fertilizer was selected for bacterial assemblage according to the ecological function and adaptation to the surrounding environment [83].

In the meantime, these bacteria negatively responded to soil EC, which explained their low abundances in LCF, despite a similar C/N ratio compared to 2/4 CN + 2/4 MN. Although previous studies reported the existence of *Flavobacterium*, *Citrobacter* and *Stenotrophomonas* in salinized conditions in the Qinghai-Tibet Plateau [84], or as a dominant genus in salinized soil [85,86], the results in this study suggested increased reproduction of these bacteria in non-salinized soil. *Aeromonas* and *Pseudochrobactrum,* in relation to soil EC and C/N ratio, were seldomly reported.

Organic materials added to the soil ecosystem could induce the carbon priming effect [78]. The selected bacteria involved in organic matter decomposition and nutrient cycling may play an important role during the process. The abundances of these bacteria did not necessarily increase after organic material addition. They were also affected by other factors, such as soil EC. In future investigations, the exogenous addition of these bacteria for regulation of decomposition should consider other possible restraining factors in agricultural ecosystems.

### 4.4. Decisive Factors in Shaping Bacterial Community

**Figure 5:**
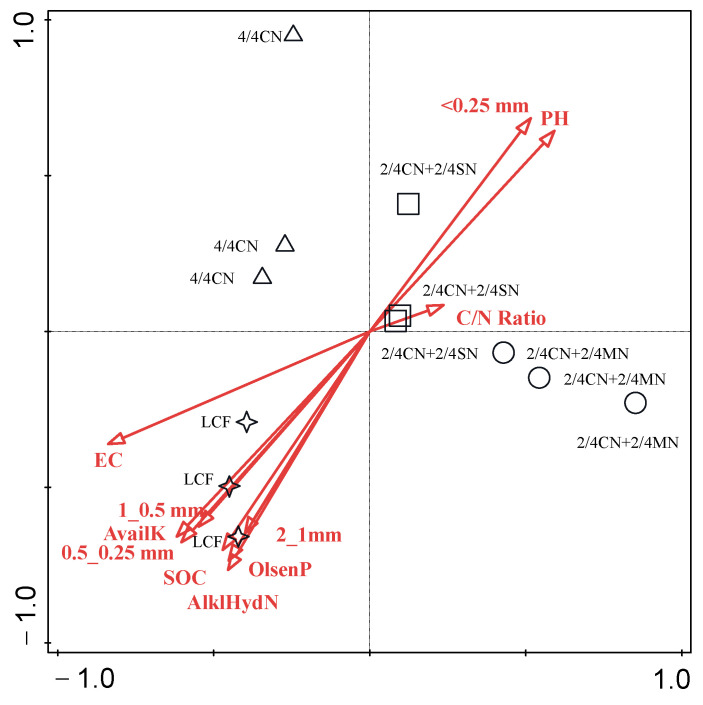
Redundancy analysis of the relationships between soil properties and soil bacterial on genus level. CN: chemical nitrogen; MN: swine manure nitrogen; SN: maize straw nitrogen; LCF: local conventional fertilization.

Decisive factors that have shaped the bacterial community after fertilizer input reduction were analyzed via RDA (Figure 5). The first two components explained a total of 76.59% (PC1 of 57.49% and PC2 of 19.11%) of the variance in the bacterial community among all treatments. Soil EC (*p* = 0.002), Olsen P (*p* = 0.006) and C/N ratio (*p* = 0.064) of input fertilizer were the decisive factors that contributed 43.8, 19.9 and 6.8% of the variance, respectively. Soil bacterial community is often in a state of dynamic change that is simultaneously affected by multiple factors, such as environmental conditions, plant root exudate and anthropogenic activity [17,55]. For agricultural land, fertilization regimes promoted the deterministic process of bacterial community via soil characteristics, such as EC, pH, nutrient availability, C availability and C/N/P ratio [17,18,55]. In the present study, soil EC was the dominant factor in influencing bacterial community, suggesting that osmotic stress due to soil salinization from continuous fertilizer over-application was the limiting factor for bacterial reproduction. Similar results of soil EC as the key factor driving the change in bacterial composition were observed in Songnen Plain [87] and in greenhouse vegetable production by Shen et al. [55] as well. C, N and P also played an important role in influencing bacterial community for their provision of basic energy and nutrients. Higher C/N ratio from organic material input supplied sufficient C for structural cellular materials, and adequate P input provided P-rich ribosomes for microbials [17,23]. The results above reiterated the necessity to reduce fertilizer application rate and the importance of organic material substitution for chemical fertilizer in regulating bacterial community and functionality.

It is worth noting that certain bacteria can be regulated by more than one soil characteristic at the same time. To add core bacteria in regulating soil ecosystems in the future, soil conditions in the target area should be taken into consideration.

### 4.5. Reduced-Fertilizer Input Affected Bacteria-Mediated N-Cycling Functioning

**Figure 6:**
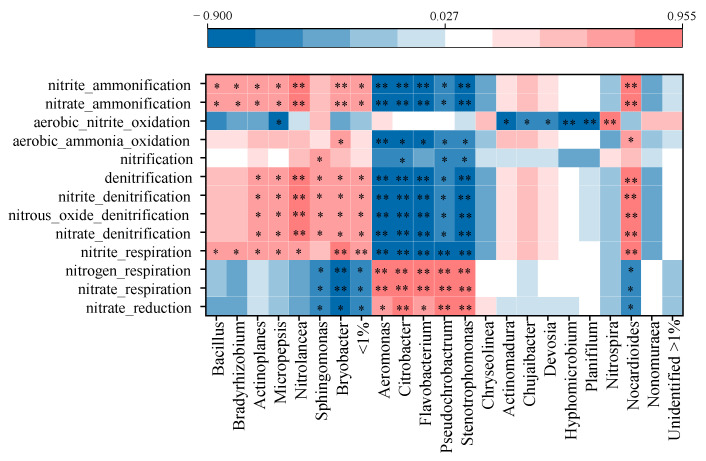
Spearman correlation analysis between N-cycling functions and bacterial community on genus level. * indicates significant correlation at *p* < 0.05, while ** indicates extremely significant correlation at *p* < 0.01.

As an important component in soil N cycling, the bacterial community connected soil function and plant productivity under specific environmental conditions in agricultural ecosystems [88]. Exogenous input, for instance, chemical fertilizer and organic materials, can shift bacterial community and their subsequent functional behavior [87]. Spearman correlation analysis revealed specific bacterial genera that show significant correlations with N-cycling functions (Figure 6). Each function was subjected to both promoting and restraining effects from relevant genera. The relative abundance of the function was a comprehensive result of these genera functioning together, such as ‘nitrate reduction’ abundance having negative correlations with *Bryobacter* and *Sphingomonas* but positive correlations with *Aeromonas*, *Citrobacter*, *Flavobacterium*, *Pseudochrobactrum* and *Stenotrophomonas* abundances. Similar results were also observed for ‘nitrate respiration’ and ‘nitrogen respiration’, indicating functional redundancy of distinct bacteria encoding the same N-cycling functions, and the same bacteria involved in distinct N-cycling functions [89,90]. Meanwhile, these cooperative and competitive relations between bacterial genera in performing N-cycling functions (nitrification and denitrification) were reported in previous studies as well [90].

Fertilization regimes changed bacterial abundances, which were then then reflected in N-cycling functioning of bacterial community. In this study, fertilizer input reduction with organic N substitution increased *Aeromonas*, *Citrobacter*, *Flavobacterium*, *Pseudochrobactrum* and *Stenotrophomonas* abundances, which had positive correlations with ‘nitrate reduction’, ‘nitrate respiration’ and ‘nitrogen respiration’. Nitrate reduction was in the process of respiration, which included dissimilatory nitrate reduction and N assimilation into biomass [90]. Dissimilatory nitrate reduction was prone to happen in a high C/N ratio [91], which was similar to the result in this study. Fertilizer input reduction with organic N substitution restrained functions related to denitrification and nitrification processes, which were positively correlated with abundances of bacteria genera of *Actinoplanes*, *Micropepsis*, *Nitrolancea* and *Bryobacter* that were enriched in LCF treatment with excessive fertilizer application. The nitrification process converts nitrogen-based fertilizers to nitrate that has a key role in N leaching, while the denitrification process loses N to atmosphere [89]. Both nitrification and denitrification accelerated N loss and decreased N use efficiency in LCF.

The results of this study suggested that N-cycling functioning of bacterial community indicated that fertilizer input reduction in combination with organic matter has potential in decreasing N loss and increasing N-use efficiency. This is supported by previous studies. Straw turnover or slow-releasing fertilizer (including organic fertilizer that requires microbial degradation and mineralization) can decrease nitric oxide emission from denitrification in farmland [63,92]. Zhao et al. [93] also reported that excessive N application in grain fields accelerated N loss via leaching or to the atmosphere based on a meta-analysis using 1174 paired observations from 69 publications. Sofo et al. [88] stated that organic inputs in combination with reduced fertilizer input could decrease N loss via greenhouse gas emission and leaching.

## 5. Conclusions

Fertilizer input reduction from conventional fertilization prevented soil acidification and salinization and increased soil bacterial richness in the greenhouse in intensive vegetable production. Reduced-fertilizer input with 50% substitution of swine manure or maize straw improved soil quality and altered N-cycling functioning with decreased nitrification and denitrification, observed after nine years, and is, therefore, recommended. Fertilization regimes shifted bacterial community via decisive factors of soil EC, Olsen P and C/N ratio of input fertilizer. Keystones of the bacterial community were responsive to altered soil properties and then the N-cycling functions, such as *Flavobacterium*, *Aeromonas*, *Citrobacter*, *Pseudochrobactrum* and *Stenotrophomonas,* that were enriched in treatments with low soil EC and high C/N ratio. In view of N-use efficiency and environmental pollution, the results of this study stated the importance of reducing fertilizer input in intensive greenhouse vegetable production in China, with a better option of organic materials in combination with chemical fertilizer.

This study focused on the belowground bacterial community, with soil samples taken at one time, more sampling on large spatial and temporal scales, along with aboveground plant productivity, N uptake and loss needed to further explore bacterial functioning in an environmental-friendly and sustainable agricultural ecosystem.

## Figures and Tables

**Table 1:** Annual NPK input of all treatments from 2009 to 2018.

Treatment	Chemical Fertilizer (kg ha^−1^)			Swine Manure (kg ha^−1^)			Maize Straw (kg ha^−1^)		
	N	P	K	N	P	K	N	P	K
2/4 CN + 2/4 MN	525	51	661	525	178	273	0	0	0
2/4 CN + 2/4 SN	525	168	234	0	0	0	525	61	700
4/4 CN	1050	229	934	0	0	0	0	0	0
LCF	1200	393	747	900	397	773	0	0	0

CN: chemical nitrogen; MN: swine manure nitrogen; SN: maize straw nitrogen; LCF: local conventional fertilization.

**Table 2:** Soil physicochemical properties of all treatments in 2018.

Treatment	EC (μs cm^−1^)	pH	Total N (g kg^−1^)	Alkali-Hydrolysable N (mg kg^−1^)	Olsen P (mg kg^−1^)	Available K (mg kg^−1^)	SOC (g kg^−1^)	Aggregate 2–1 mm (%)	Aggregate 1–0.5 mm (%)	Aggregate 0.5–0.25 mm (%)	Aggregate <0.25 mm (%)
Before planting	185	8			6.2	98.2	5.3				
2/4 CN + 2/4 MN	414 c	7.5 a	1.5 b	80.2 b	131.3 b	355.0 b	13.1 bc	3.39 b	6.40 b	8.25 b	72.82 a
2/4 CN + 2/4 SN	454 c	7.5 a	1.8 b	82.0 b	104.7 c	256.6 b	16.2 b	3.25 b	5.74 b	10.61 b	70.91 a
4/4 CN	820 b	7.5 a	0.9 c	39.0 c	95.6 c	394.5 b	6.0 c	2.85 b	6.31 b	9.16 b	73.83 a
LCF	1261 a	5.4 b	5.5 a	273.7 a	300.3 a	933.3 a	54.0 a	6.80 a	14.75 a	18.36 a	52.85 b

Numbers followed by different letters are significantly different (*p* < 0.05). SOC: soil organic C; CN: chemical nitrogen; MN: swine manure nitrogen; SN: maize straw nitrogen; LCF: local conventional fertilization.

**Table 3:** Richness and diversity estimates of bacterial community for all treatments in 2018.

	Variable		Richness		Diversity	
Treatment		OTU Observed	Chao1	ACE	Shannon	Simpson
2/4 CN + 2/4 MN		1879 a	2784 a	3128 a	5.12 a	0.035 a
2/4 CN + 2/4 SN		1959 a	2758 a	2951 a	5.85 a	0.012 b
4/4 CN		2115 a	2906 a	3060 a	6.01 a	0.005 b
LCF		1312 b	1626 b	1623 b	5.55 a	0.010 b

OTU: operational taxonomic units (97% similarity). CN: chemical nitrogen; MN: swine manure nitrogen; SN: maize straw nitrogen; LCF: local conventional fertilization. Numbers followed by different letters are significantly different (*p* < 0.05).

## Data Availability

Gene sequences of all samples are deposited in the National Center for Biotechnology Information (NCBI) Sequence Read Archive (PRJNA818078).
